# Immunopathophysiology and clinical impact of uveitis in inflammatory rheumatic diseases: An update

**DOI:** 10.1111/eci.13572

**Published:** 2021-05-05

**Authors:** Elvis Hysa, Carlo Alberto Cutolo, Emanuele Gotelli, Greta Pacini, Carlotta Schenone, Elke O Kreps, Vanessa Smith, Maurizio Cutolo

**Affiliations:** ^1^ Laboratory of Experimental Rheumatology and Academic Division of Clinical Rheumatology Department of Internal Medicine Italy – IRCCS Rheumatology Unit San Martino Polyclinic University of Genoa Genoa Italy; ^2^ Ophthalmology Clinic DiNOGMI IRCCS Ophthalmology Unit San Martino Polyclinic University of Genoa Genoa Italy; ^3^ Department of Ophthalmology Ghent University Hospital Ghent Belgium; ^4^ Department of Internal Medicine Department of Rheumatology Ghent. University Hospital Ghent University Ghent Belgium; ^5^ Unit for Molecular Immunology and Inflammation VIB Inflammation Research Center (IRC) Ghent Belgium

**Keywords:** autoimmune rheumatic diseases, behçet's disease, connective tissue diseases, sarcoidosis, spondyloarthritis, uveitis

## Abstract

**Background:**

Uveitis is one of the most frequent ophthalmologic manifestations in rheumatology. Uveal inflammation can underlie a systemic inflammatory rheumatic disease (SIRD) in approximately 30% of cases with a significant burden on the quality of life since it represents a cause of blindness in up to 20% of cases in Western countries.

**Methods:**

In this review, we provide a comprehensive overview of the pathophysiology of uveitis associated with SIRDs. According to our literature survey on the epidemiology of uveitis among SIRDs, spondyloarthritides, Behçet's disease and sarcoidosis get the major impact.

**Results:**

In Behçet's uveitis, the key players are highly polarized Th1 and Th17 lymphocytes, natural killer T cells and γδ T cells. All contribute to a great destructive inflammatory environment with the most serious visual damage resulting from the involvement of the posterior segment of the eye. In contrast, spondyloarthritides‐related uveitis derives from a complex interaction between genetic background and extra‐ocular inflammatory mediators originating from enthesitis, arthritis, psoriatic lesions and microbiome pro‐inflammatory alterations. In such conditions, the immune infiltration of CD4+ T cells, Th17 and natural killer cells along with pro‐inflammatory cytokines, TNF‐α among all, leads to intraocular inflammation. Lastly, granuloma formation represents the primary hallmark lesion in sarcoid uveitis. This suggests a profound link between the innate system that mainly recruits activated macrophages and adaptive system involving by Th1, Th17 and Th17.1 cells.

**Conclusions:**

Awareness among rheumatologists of a potential severe ocular involvement generates new insights into targeted therapeutic approaches and personalized treatments for each patient.

## 1 INTRODUCTION

In normal conditions, the eye is characterized by a privileged immune state, which is set to provide protection against local inflammation and to minimize the risk of visual impairment.^1^ Despite the presence of strong blood‐tissue barriers formed by endothelial cell tight junctions, innate and adaptive immune cells and molecules can still gain access to the intraocular microenvironment.^2^ This process is particularly pronounced in systemic inflammatory rheumatic diseases (SIRDs), when effector leucocytes are already activated, the regulatory immune response is impaired and the damaged endothelium represents an entry gate for cytokines and inflammatory cells, eventually resulting in tissue damage through different mechanisms.^3^, ^4^

Uveitis is the most common ophthalmologic finding in the rheumatologic practice with a prevalence of 5.4/1000 individuals, according to the geographic area.^5^ It is defined as inflammation of the uvea, the vascular layer of the eye that includes iris, ciliary body and choroid.^6^ The inflammatory process can involve other ocular structures such as the retina, sclera, cornea, vitreous and optic nerve with a broad spectrum of signs and symptoms severity according to the extent of the inflammatory process.

A prompt treatment is required since uveitides represent 10%‐20% of causes of preventable blindness in the Western countries.^5^ Several causes of uveitis have been identified: infectious, immune‐mediated, masquerade syndromes and drug‐related forms.^7^ Nonetheless, a variable percentage of cases remains idiopathic.^7^ Data from multiple retrospective analysis of records of patients diagnosed with uveitis after a detailed ophthalmologic work‐up show that SIRDs can cover approximately 30% of cases. Particularly, seronegative spondyloarthritis, Behçet's disease (BD) and sarcoidosis display the major epidemiological weight^7^, ^8^, ^9^, ^10^, ^11^, ^12^, ^13^ (Table 1).

**TABLE 1:** Epidemiological weight of SIRD‐associated uveitides among the causes of uveitis in different cohorts of patients worldwide

	Percentage distribution of uveitis by associated SIRDs worldwide						
Disease	Australia^13^ (%) (n = 1236)	Italy^8^ (%) (n = 1064)	France^7^ (%) (n = 912)	China^9^ (%) (n = 823)	Japan^10^ (%) (n = 750)	United States^12^ (%) (n = 491)	Tunisia^11^ (%) (n = 472)
Behçet's disease	1.8%	5.3%	8.2%	2.8%	4.4%	n.a	12.2%
Ankylosing spondylitis	3.6%	n.a	n.a	5.8%	n.a	1%	1.7%
IBD‐related spondyloarthropathies	0.6%	n.a	n.a	n.a	n.a	1.2%	0.8%
Psoriatic arthritis	0.1%	n.a	n.a	1.1%	n.a	0.2%	0.8%
Reactive arthritis	0.3%	n.a	n.a	n.a	n.a	0.2%	0.2%
Sarcoidosis	3.5%	2.5%	17.1%	1.4%	6.1%	6.7%	1.7%
Vogt‐Koyanagi‐Harada syndrome^a^	n.a	2%	2.6%	n.a	4.1%	n.a	n.a
Rheumatoid arthritis	0.3%	n.a	n.a	0.003%	0.2%	n.a	0.2%
Juvenile idiopathic arthritis	0.6%	n.a	n.a	1.1%	0.2%	n.a	0.6%
Idiopathic	60.2%	26%	46.9%	n.a	40.7%	32.2%	35.2%
HLA‐B27‐associated uveitis^b^	11.2%	5.3%	16.7%	n.a	1.8%	6.7%	1%
Others^c^	17.8%	58.9%	8.5%	87.8%	42.5%	51.8%	45.6%

Abbreviations: HLA‐B27, human leucocyte antigen B27; IBD, inflammatory bowel disease; n.a, Not assessed; SIRDs, systemic inflammatory rheumatic diseases.

^a^ Vogt‐Koyanagi‐Harada disease was included in the list because of its immune‐mediated origin despite not being a rheumatologic condition.

^b^ In some studies, the seronegative spondyloarthropathy underlying HLA‐B27 positivity was not specified and patients were classified as generally affected by ‘HLA‐B27 associated uveitis’.

^c^ Including infectious, immune‐mediated, neoplastic, drug‐related and post‐surgical causes.

The most common symptoms are blurred vision, eye pain and sensitivity to light.^14^ The signs of uveitis are very numerous and can include ocular redness, cells and flare in the anterior chamber, keratic inflammatory precipitates, iris inflammatory nodules, iridolenticular synechia and specific changes of the retinal, choroidal and vascular tissues. However, many others subtle signs can be present, and symptoms may range from foreign body sensation to extreme pain and from floaters to profound visual loss.

The anatomical classification of uveitides categorizes them into four groups: anterior uveitis, intermediate uveitis, posterior uveitis and panuveitis.^5^ This categorization is based on the primary location of the inflammatory activity and not on the occurrence of complications (eg an anterior uveitis with secondary cystoid macular oedema continues to be an anterior uveitis despite complications in the posterior segment).^5^

In anterior uveitis, which accounts for 75%‐90% of cases, the primary site of inflammation is the anterior chamber and retrolental space (iridocyclitis), whereas the vitreous cavity, posterior ciliary body and pars plana are primarily affected in intermediate uveitis. By contrast, posterior uveitis mainly damages the retina and/or choroid, whereas in panuveitis, the inflammation is diffuse without a predominant location.^5^

The pathogenetic alterations leading to SIRD‐associated uveitis are still poorly understood, but an aberrant inflammatory response involving both adaptive and innate arms of the immune system seems to be involved (Table 2).

**TABLE 2:** Summary of the immunopathogenesis of the major systemic inflammatory rheumatic disease‐related uveitides

		Immunopathogenesis of uveitis associated with inflammatory rheumatic diseases			
Disease	Main involved cytokines	Main cellular adaptive effectors	Main cellular innate effectors	Ocular manifestations	Prognosis
Behçet's disease	IL‐15, IFN‐γ, TNF‐α, IL‐18, IL‐6, IL‐8.^39^	CD8+ T cells, Th1, Th17, Th22 NKT cells.^35^, ^42^, ^44^ T regs deficiency^41^	Neutrophils, dendritic cells, γδ T cells^37^, ^40^, ^50^	Severe panuveitis with hypopyon in 25% of cases, retinal peri‐phlebitis^54^	Poor. Vision loss can occur in up to 25% of cases^54^
HLA‐B27 spondyloarthropathies	IL‐2, IL‐6, IFN‐γ, TNF‐α.^75^	CD4+ T cells, Th1, Th17. ^77^ Quantitative Tregs deficiency.^77^	NK cells.^64^ Role of resident IL‐23 R γδ T cells?^72^	Acute anterior uveitis: mainly unilateral but can be also bilateral^59^	Usually good but relapses and complications may occur^78^
Sarcoidosis	TNF‐α, IL‐2, IFN‐γ.^99^ SAA^91^	CD4+ Th1 cells,^99^ CD4+ Th17, CD4+ Th 17.1.^86^, ^88^ Qualitative deficiency of Tregs^90^	Macrophages^85^	Acute/chronic uveitis, intermediate uveitis, multifocal choroiditis, retinal vasculitis, optic disc swelling^97^	Intermediate to severe. In up to 45% of cases, vision loss can occur^94^

Abbreviations: CD, cluster of differentiation; HLA‐B27, human leucocyte antigen B27; IFN‐γ, interferon gamma; IL‐, Interleukin‐; NK, natural killer cells; NKT, natural killer T cells; SAA, serum amyloid A; Th, T‐lymphocyte helper; TNF‐α, tumour necrosis factor alpha; Tregs, T regulatory cells; γδ T cells, gamma delta T cells.

## 2 PATHOGENETIC INSIGHTS FROM EXPERIMENTAL AUTOIMMUNE UVEITIS IN THE ANIMAL MODELS

Over the last decades, the murine model of experimental autoimmune uveitis (EAU) has significantly contributed to the understanding of the immunological alterations leading to intraocular inflammation.^3^

Ocular inflammation can be induced by immunization with retinal antigens: S‐antigen (Ag‐S) has been used in rats, whereas rhodopsin and interphotoreceptor retinoid‐binding protein (IRBP) have been studied in mice.^15^

After about ten days post immunization, infiltration of immune cells can be observed in the retina and choroid of B10.RIII mice, the most susceptible mouse strain known to develop EAU.^16^ Increased concentrations of interleukin (IL)‐2 and interferon gamma (IFN‐γ) have been detected in draining lymph nodes at weeks 1 and 2, suggesting a main T helper (Th) 1 response. Conversely, Th 2‐type responses were associated with the resolution phase of EAU.^17^

Additionally, IL‐17‐producing CD4+ T cells, defined Th17 lymphocytes, represent immune cells associated with the induction of EAU. Indeed, IL‐17 knockout mice (IL‐17 a ‐/‐) showed significantly reduced severity of uveoretinitis compared with wild‐type mice.^18^ The importance of autoreactive T cells in uveal inflammation is also evidenced by studies which have detected a response of T lymphocytes in patients with BD towards heat shock proteins (Hsp), highly conserved molecules in mammalian cells.^19^

Particularly, it has been shown that administration of 65 kilodalton heat shock protein (Hsp65) in mice was associated with the expansion of CD4+ IFN‐γ + and CD4+IL‐17 + T cells in the draining lymph nodes aggravating EAU.^20^

However, other cellular effectors have been investigated in the pathogenesis of uveitis. Recently, cluster of differentiation (CD)4+CD25+ regulatory T (Treg) cells have been shown to play a key role in the regression of EAU displaying a pivotal immunomodulatory role.^21^ Indeed, in the peripheral blood of BD patients, increased concentrations of CD4+CD25+ Treg cells have been detected in subjects with active disease compared with BD patients in remission suggesting a potential attempt of the immune system to counterbalance active inflammation.^22^ Despite this finding has not been confirmed by *Gündüz et al*,^23^ who have detected a decreased number of peripheral Tregs in active BD, the involvement of CD4+CD25+ Tregs in uveitis resolution appears compelling both because decreased percentages of Treg cells have been reported to be a predictive marker of ocular attack^24^ in BD patients and because this suppressor cell line has been shown to downgrade EAU in the rat.^25^

Aside from cellular components, several pro‐inflammatory molecules are associated with EAU development: particularly, adhesion molecules are associated with migration of inflammatory cells into the eye. Indeed, increased expression of intercellular adhesion molecule‐1 (ICAM‐1) and very‐late antigen 4 (VLA‐4) has been demonstrated in mice after immunization with IRBP, evidenced by the inhibition of EAU development when antibodies against ICAM‐1 and VLA‐4 were administered.^26^

Immune cell migration might represent the first pathogenetic hit in uveitis development. Inflammatory cell infiltration of the retina has been shown to be prevented in experimental mouse models treated with fingolimod, a drug inhibiting T‐cell migration by sequestering these cells in secondary lymphoid organs.^27^ Indeed, fingolimod has shown to suppress, in rats immunized with Ag‐S, the incidence and intensity of EAU and decrease the serum concentrations of antibodies directed against Ag‐S and antigen‐specific lymphocyte proliferation with a histologic evidence of disease suppression.^28^

Considering the pro‐inflammatory cytokines leading to EAU, IL‐6 and tumour necrosis factor alpha (TNF‐α) are among of the major protagonists. This statement is indirectly proven, on the one hand, by the efficacy of anti‐IL‐6 receptor monoclonal antibody in suppressing ocular inflammation in mice and, on the other hand, by treatment effectiveness of etanercept, infliximab and adalimumab, TNF‐α antagonists, in patients with uveitis.^29^, ^30^

The pathogenetic events of uveitis appear to be finely orchestrated by different arms of the immune system. Even though no single animal model can reproduce the full complexity of the human eye disease, these studies might provide important hints about the key players driving inflammatory ocular disease.

In the next paragraphs, the pathophysiological mechanisms of uveitis occurring in the major inflammatory rheumatic diseases of the adult will be elucidated in light of the most recent evidence.

## 3 UVEITIS IN BEHÇET’S DISEASE

Behçet's disease (BD) is a rare and severe multisystemic autoimmune disease, characterized by recurrent oral aphthous ulcers, genital ulcers, skin lesions and both anterior and posterior uveitis.^31^ It is classified as a systemic vasculitis and may affect every tissue and organ of the body: joints, gastrointestinal tract, nervous system and others.

In BD, the predominant ocular immune infiltration is composed of T cells, suggesting that the related uveitis is mainly a T cell–mediated autoimmune disease, elicited by a hypersensitivity response towards an unknown antigen in a patient with genetic or epigenetic predisposition.^32^

The hypothesized triggering antigens appear to be microbial antigens (herpes simplex virus‐1, Borrelia burgdorferi, Helicobacter pylori), heat shock protein 65 (HSP65), the interphotoreceptor retinoid‐binding protein (IRBP) and retinal S autoantigen.^33^

**FIGURE 1:**
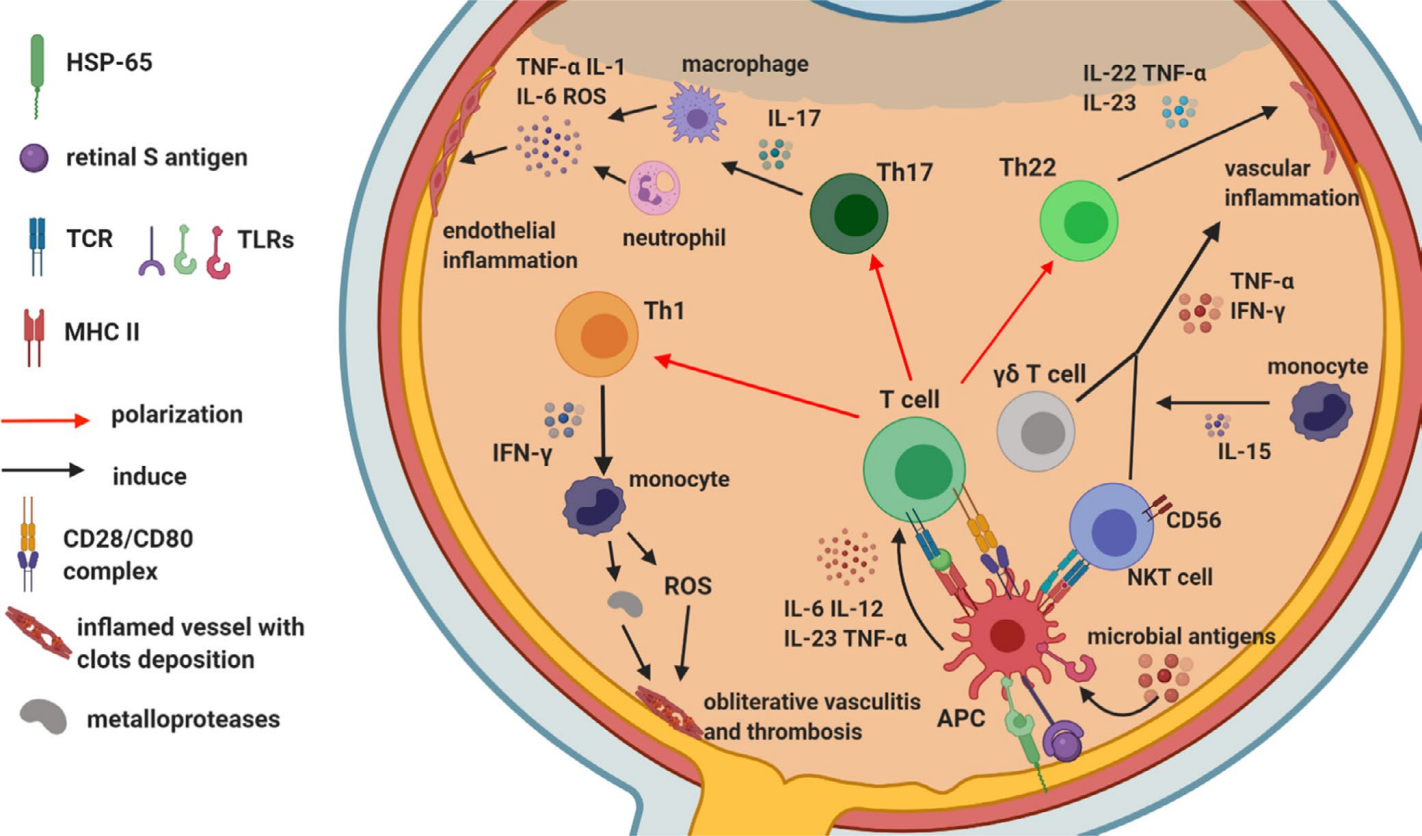
Immune‐mediated mechanisms in Behçet's uveitis. HSP‐65, Heat shock protein 65; TCR, T‐cell receptor; TLR, toll‐like receptor; MHC‐II, major histocompatibility complex II; CD, cluster of differentiation; APC, antigen‐presenting cell; IL‐, interleukin‐; TNF‐α, tumour necrosis alpha; IFN‐γ, interferon gamma; Th, T helper cell; ROS, reactive oxygen species; NKT, natural killer T cells

The genetic background involves the presence of HLA‐B51, HLA‐A26, polymorphisms in TNF‐α gene, IL‐10 gene, IL‐23R/IL‐12RB2 gene and microRNAs (miR) downregulation (in particular miR‐155)^34^ (Figure 1).

In Behçet's uveitis (BU), CD8+ T cells and natural killer T (NKT) cells, unconventional T cells expressing both markers of NK cells such as CD56, αβ T‐cell receptor (TCR) and CD3 T‐cell co‐receptor, have been detected in much higher concentrations in the aqueous humour, whereas CD4+ T cells appear to be the predominant infiltrating cells in patients with other immune‐mediated uveitides^35^ (Figure 1).

The predominance of CD8+ T cells and NKT cells in the intraocular infiltrating cell population in active BU is in line with the unique increase in aqueous IL‐15 levels.^36^ IL‐15 is involved in the development and survival of immune effector cells, such as natural killer (NK) cells, NKT cells and CD8+ T cells, and contributes to homeostasis and to the activation of gamma delta (γδ) T cells^37^ (Figure 1). The latter are T cells expressing a TCR composed of one γ‐chain and one δ‐chain having features of both innate and adaptive immunity and exhibiting a potent cytotoxic effector activity.^38^

Compared with other immune‐mediated uveitides, in active BU patients, higher aqueous concentrations of IFN‐γ and TNF‐α and lower levels of IL‐4 have been measured.^39^

Interestingly, an extremely pronounced polarization towards Th1 phenotype has been suggested in patients with BU: CD8+CD56+ T cells and CD56+ γδ T cells are considered to be the primary cells responsible for IFN‐γ secretion. This cytokine, together with TNF‐α, exerts deleterious inflammatory effects on vascular endothelial cells through the production of nitric oxide (NO).^40^

Notably, IL‐10 has never been detected in BU and deficient immunoregulatory processes have been hypothesized in patients with BU as we mentioned in the previous paragraph. Interestingly, patients treated with infliximab, a TNF‐α‐antagonist, showed a higher expression of forkhead box P3 (FOXP3), a Treg‐specific marker, on CD4+ lymphocytes compared with patients treated with colchicine or cyclosporine did not experience any subsequent episodes of acute uveitis.^41^ Conversely, in patients with a low population of Foxp3+ cells, a higher frequency of ocular inflammatory episodes was observed.^41^

Besides Th1 polarization, accumulating evidence suggests that also Th17 and Th22 lymphocytes play an important role in BU (Figure 1). In patients with active uveitis, IL‐17 concentrations are elevated in peripheral blood and in the aqueous humour: the major identified producers of IL‐17 are memory T cells (CD4+ CD45RO+) and γδ T cells.^42^ In another study, serum levels of IL‐17, IL‐23 and IFN‐γ were significantly higher in BD patients with active uveitis than in BD patients without uveitis or healthy controls.^43^

Conversely, Th22 CD4+ lymphocytes are a novel subset effector Th cells mainly producing IL‐22 and TNF‐α but no other Th cytokines such as IFN‐γ (Th1), IL‐4 (Th2) or IL‐17 (Th17)^44^ (Figure 1). Th22‐type T‐cell clones have been detected in high amounts from the aqueous humour of active BU patients, and IL‐22 levels were found to be correlated with the severity of retinal vasculitis.^45^

An aberrant activation of the innate immune system has been highlighted as well in BU pathogenesis. Interestingly, increased serum concentrations of alarmins, proteins capable of initiating the innate immune response after release from cell necrosis, have been detected in BD patients and bacterial sensing mechanisms have been theorized as important pathogenetic processes.^46^ As a matter of fact, toll‐like receptor (TLR) 2 and TLR4 expression in monocytes from BD patients have been detected as constitutively increased,^47^ whereas another paper has shown an increase in TLR6 expression of granulocytes from BD patients after stimulus with Streptococcus sanguinis or HSP‐60 compared with rheumatoid arthritis patients and healthy controls.^48^

Along with neutrophil and monocytes hyperactivity,^46^ dendritic cells (DCs), the most potent antigen‐presenting cells, play a crucial role in perpetuating inflammation in BU.^49^ High expression of costimulatory molecules and MHC class II in BD patients in remission suggests that DC maturation is related to the chronicity and recurrence of uveitis.^49^

Phenotypical analysis of DC subsets has been performed in BD and in peripheral blood, showing lower plasmacytoid DC percentages in BD patients than in healthy controls.^50^ This result indicates the probable migration of these cells and their accumulation in inflamed tissues and suggests active participation of this cellular subset in the pathogenesis of BD.

Eventually, pro‐inflammatory cytokines play a role in the priming of neutrophils which in turn aggregate in inflammatory infiltrates forming the hypopyon and activating each other through IL‐18 secretion.^51^ Consequently, the reactive oxygen species (ROS) generated by activated neutrophils act as final effectors of the vascular endothelial cell dysfunction and thrombosis^52^ (Figure 1).

The eye is the most commonly involved organ in BD, within 2‐4 years of its onset, especially in human leucocyte antigen B51‐positive (HLA‐B51+) patients.^53^ Ocular manifestations affect up to 70% of patients with BD with severe vision loss occurring in up to 25% of cases.^54^ Ocular symptoms include periorbital pain, redness, photophobia and blurred vision. The recurrent attacks of intraocular inflammation may result in progressive, ischaemic damage of the retina, often causing irreversible visual loss.^54^

Generally, the initial inflammatory activity is located in the anterior segment of one eye and later on tends to involve the posterior segment of both eyes. In the majority of cases, it presents as panuveitis, which tends to be more recurrent and sight‐threatening compared with HLA‐B27‐related uveitides.^53^ Cystoid macular oedema is one of the most common complications but also cataract, posterior synechia, iris bombé and angle closure may all develop.^54^

Retinal peri‐phlebitis is one of the most common ocular manifestations often associated with periarteritis, whereas anterior segment inflammation includes hypopyon formation, a sedimentation of white blood cells, in up to 25% of cases. Uveitis can be severe and visual loss may also develop as a result of retinal vasculitis and its complications, such as macular oedema and others.^54^

In summary, the mechanisms leading to BU involve a complex orchestration of different mediators belonging to both innate and adaptive immune systems. Interestingly, the unique intraocular cellular and cytokine environment in BU may reflect the more recurrent and greater destructive nature of BU compared with other uveitides.

Further research into the immunopathogenic processes involved in the development of BU could define critical points in the induction of ocular inflammation and unveil new horizons about targeted therapeutic approaches as well as customized treatment in each patient.

## 4 UVEAL INFLAMMATION IN SPONDYLOARTHROPATHIES

Seronegative spondyloarthropathies (SpA) are a family of inflammatory rheumatic diseases with common clinical and aetiological features including axial and peripheral inflammatory arthritis, enthesitis, extra‐articular manifestations and a strong association with the presence of the HLA‐B27 epitope.^55^, ^56^, ^57^

Specifically, ankylosing spondylitis (AS), psoriatic arthritis (PA), inflammatory bowel disease (IBD)–associated spondyloarthropathies, reactive arthritis and undifferentiated spondyloarthropathies are incorporated in this group.^56^, ^58^

Acute anterior uveitides (AAU) occur in up to one‐third of the cases in patients affected by SpA.^59^ Their pathogenesis is incompletely understood and is attributed to the interaction between genetical background (HLA‐B27 association in particular) and external factors.^59^

In detail, different tissue‐specific impairments have been hypothesized but not fully proven.

**FIGURE 2:**
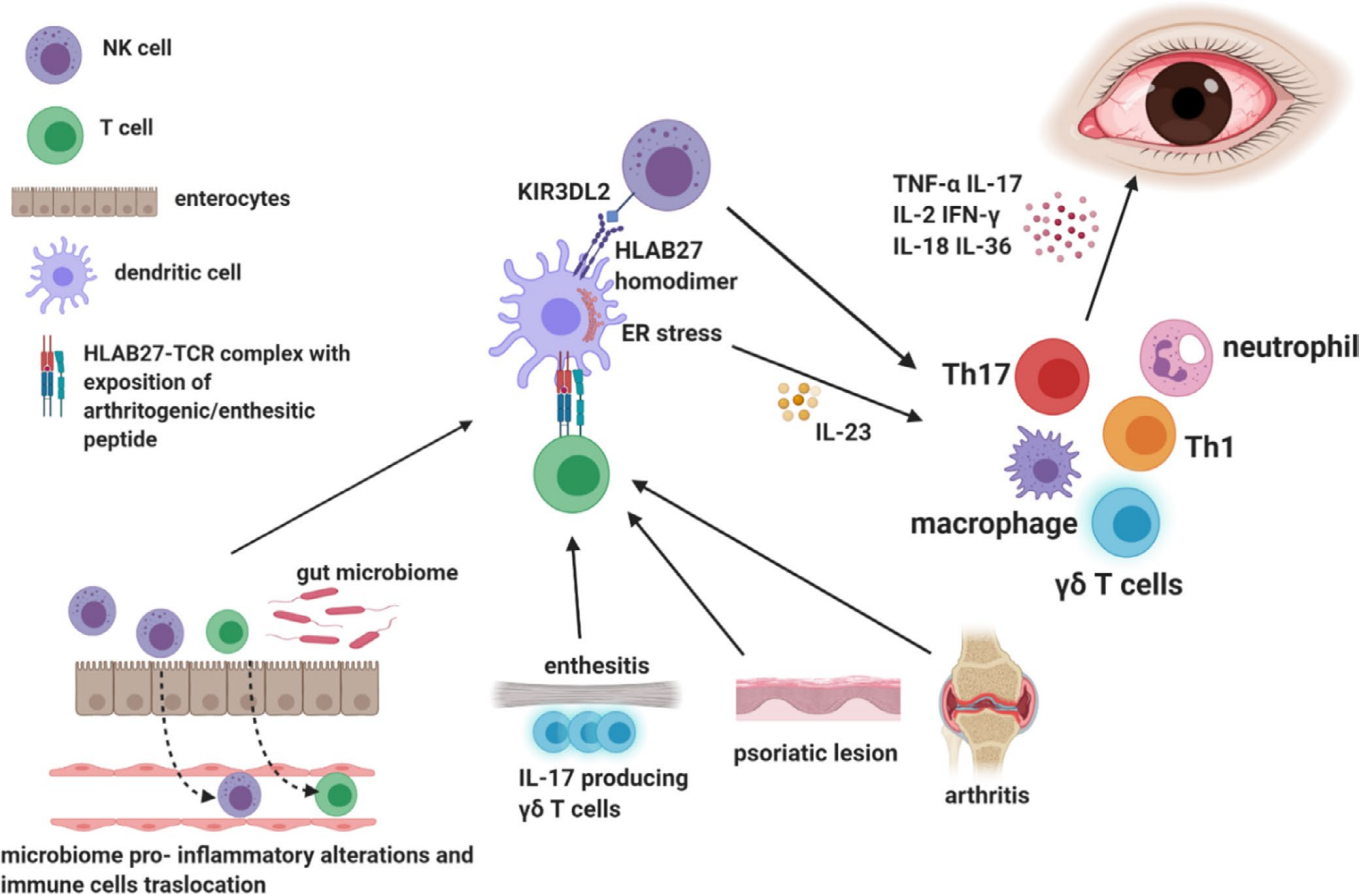
Immunological mechanisms of spondyloarthritis‐related uveitis. HLAB27, Human leucocyte antigen; TCR, T‐cell receptor; IL‐, interleukin‐; TNF‐α, tumour necrosis factor alpha; IFN‐γ, interferon gamma; Th, T helper cell; ER, endoplasmic reticulum; KIR3DL2, killer cell immunoglobulin‐like receptor 3DL2; γδ T cells), gamma delta T cells

Firstly, a potential molecular mimicry between bacterial proteins and ‘uveitic’ antigens has been theorized.^60^ Other hypothesized mechanisms are microbiome alterations,^61^ mechanical stress in the entheses, lens and ciliary body and a higher susceptibility of the uvea to endoplasmic reticulum (ER) stress response with generation of pro‐inflammatory cytokines^61^, ^62^ (Figure 2).

Lastly, an enhanced expression of mucosal and vascular adhesion molecules may allow the infiltration from immune cells from extra‐ocular sites of the body such as the gut.^61^

On the one hand, abnormal forms of HLA‐B27 might preferably present ‘uveitic’ antigens to CD4+ T cells and NK cells eliciting their autoreactivity. Consequently, inflammatory damage in the joints and in the eye is caused.^61^ On the other hand, HLA‐B27 folding alterations may be involved in the generation of an ER stress response with the inhibition of IL‐10 immunoregulatory cascade and IL‐23/IL‐17 axis activation, which represents one of the major pathogenetic fingerprints of spondyloarthropathies^62^, ^63^ (Figure 2).

Additionally, HLA‐B27 tendency to form homodimers or to be exposed on the APC as a heavy chain could aberrantly activate the innate counterpart of the immune systems through the binding with innate immune receptors such as the killer cell immunoglobulin‐like receptor 3DL2 (KIR3DL2) on NK cells, leading to inflammatory mediators’ release^64^ (Figure 2).

Furthermore, HLA‐B27 signature might alter the composition of the gut microbiome which, in turn, creates an imbalance of the immune system. This hypothesis is sustained by the animal models: HLA‐B27 transgenic mice do not develop uveitis, enthesitis or arthritis when kept in a germ‐free environment.^65^

The most accepted molecular mechanisms of the gut‐eye axis are various.

One of them is an impaired induction from gut bacteria of Treg cells expressing fork head box P3 (FOXP3+) and synthetizing IL‐10.^66^ Additionally, a potential molecular mimicry between ocular proteins (such as retinal antigen IRBP) with antigens in the gut bacteria has been described.^67^

Gut permeability in SpA is also increased because of the local inflammatory alterations of the intestine to microbial products consequently eliciting an abnormal innate or adaptive immune response in extra‐intestinal sites like the intraocular environment.^68^ Lastly, the extra‐intestinal translocation of immune cells in the eye has been proven in experimental autoimmune uveitis (EAU) in the murine model where intestinal‐derived CD45+ leucocytes have been detected in the eye.^69^

Besides B27‐association, other susceptibility genes outside the HLA are involved in antigen processing and cytokines: particularly, polymorphisms of the components of IL‐23/IL‐17 axis have been reported.^70^ In the latest years, the key role of IL‐17 and IL‐23 in inducing enthesitis, a hallmark of SpA, has progressively been heightened.^71^

This concept has been documented both by studies in mice and humans: in mice entheses, resident cells bearing the IL‐23 receptor (IL‐23R) and the γδ TCR produce IL‐17 and IL‐22, whereas, recently, it has been proven that tissue resident human enthesis γδ T cells can produce IL‐17A independently of IL‐23R transcript expression.^72^, ^73^

Interestingly, *McGonagle et al* proposed that connective tissue of uveal structures might be conceptually similar to a musculoskeletal enthesis. Indeed, elastin and type IV collagen compose the structure of tendons of the ciliary muscle.^74^ Curiously, IL‐23R‐positive resident cells have also been recently detected in the ciliary body of mice but their role is still to be understood since uveitis was not reported in this paper.

Last but not least, the cytokinic environment detected in the aqueous humour and sera of AAU patients has been a topic of interest in several studies. Despite the absence of a specific cytokinic signature for AAU, higher concentrations of aqueous IL‐1, IL‐18, IL‐36 and increased serum concentrations of IL‐2 and IFN‐γ have been detected compared with controls.^75^

Interestingly, increased levels of TNF‐α and IL‐6 have been dosed in both serum and aqueous humour in AAU compared with controls.^76^ With regard to cellular effectors of AAU, an increased blood ratio of CD4+ Th1 cells and CD4+ Th17 cells to CD4+CD25+ Foxp3 Tregs has been reported suggesting a precise T helper polarization and a deficiency of Tregs.^77^

The clinical features of AAU are typical, often with unilateral involvement, sudden onset, photosensitivity and blurred vision. Fine keratic precipitates are observed on the corneal endothelium in most cases. Anterior chamber shows intense cellularity and flare, whereas hypopyon remains a rare finding.

The prognosis of HLA‐B27‐associated uveitis is usually good in the long term, but complications and recurrences may occur.^78^ Particularly, pupil occlusion caused by a fibrinous inflammatory membrane may cause pupil block, elevated intraocular pressure and secondary glaucoma. Macular and optic nerve head involvement may also be observed.

To date, TNF‐α is still the most targeted cytokine in the treatment of SpA‐associated uveitis. Infliximab, adalimumab and certolizumab pegol, TNF antagonists, have proven their efficacy and are currently used to treat recurrent SpA‐associated uveitides.^30^

IL‐23/IL‐17 axis appears to be an appealing therapeutic target but, at the moment, secukinumab,^79^ an IL‐17 antagonist, has shown no significant effect on uveitis, whereas a phase two trial with ustekinumab, an IL‐12/IL‐23 blocker, is currently ongoing (www.clinicaltrials.gov NCT02911116).

In conclusion, AAU is the most frequent extra‐articular manifestation of SpA. The genetic background and aberrant immune response potentially starting from peripheral musculoskeletal sites or triggered by the gut microbiome are puzzles which need to be solved in order to efficiently treat or even prevent ocular disease in these patients.

## 5 SARCOID UVEITIS

Sarcoidosis is a chronic multisystemic granulomatous disorder resulting from an exaggerated cellular immune response to a variety of self or non‐self‐antigens. It is characterized by the formation of noncaseating granulomas which typically affect the lungs, intrathoracic lymph nodes, eyes and skin.^80^

The interaction between genetic susceptibility and environmental triggers (infectious, organic and inorganic agents) is thought to be at the base of disease development.

Particularly, the genetic background involves association with HLA genes and non‐HLA polymorphisms.^81^ HLA‐DR3 is associated with an increased risk in Scandinavian populations, whereas HLA‐DRB1*11:01 carries an enhanced risk in African Americans and Caucasians.^81^ Extra‐HLA investigated genes are polymorphisms in butyrophilin‐like 2 (BTLN2), an immunoglobulin participating in T‐cell activation, TNF‐α gene and annexin A11, being involved in granulomas formation.^81^

**FIGURE 3:**
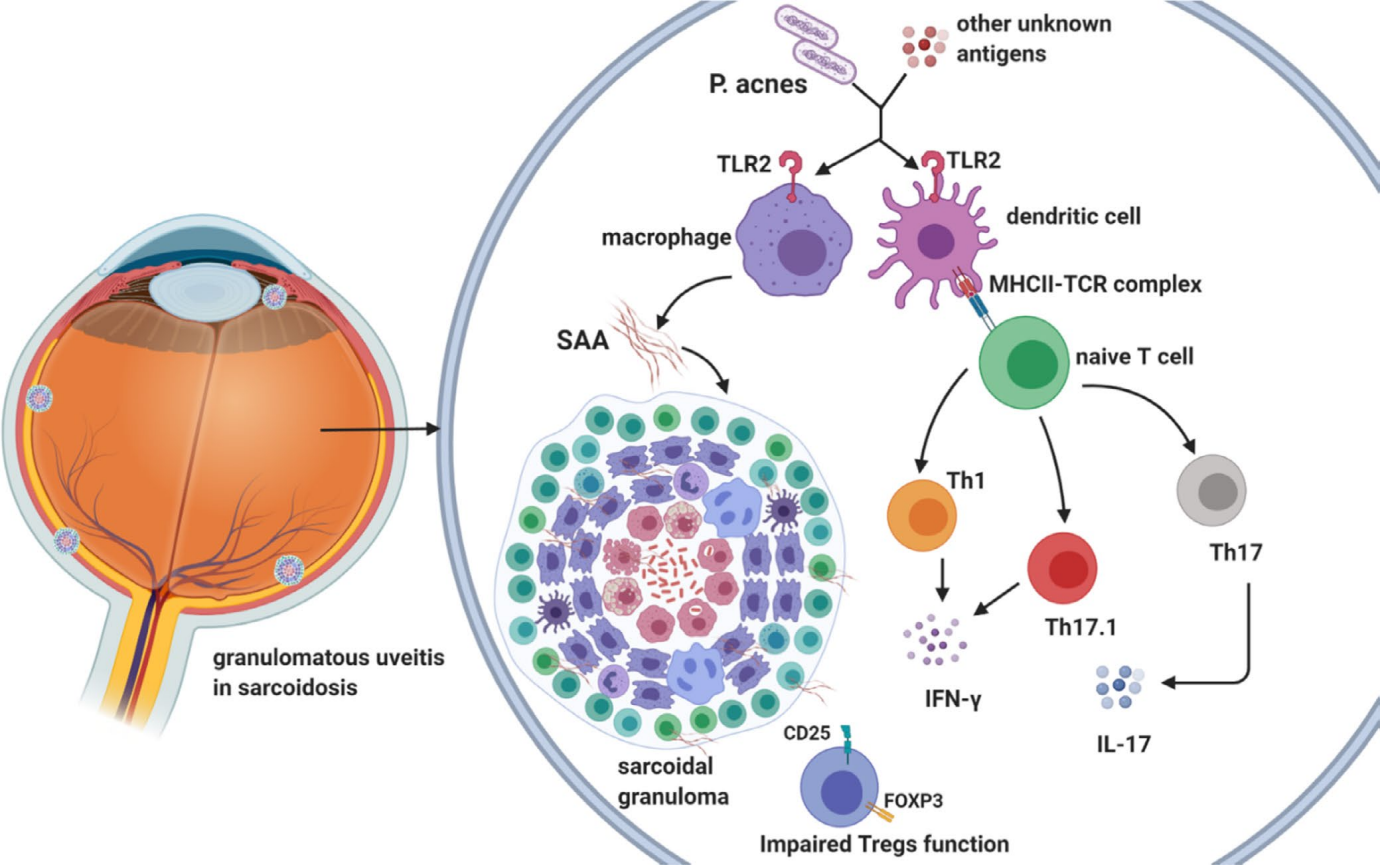
Immunopathogenesis of ocular sarcoidosis. P. acnes, Propionibacterium acnes; TLR2, Toll‐like receptor 2; MHC‐II, major histocompatibility complex 2; TCR, T‐cell receptor; Th, T helper cell; IL‐, interleukin‐; IFN‐γ, interferon gamma; CD, cluster of differentiation; Tregs, T regulatory cells; SAA, serum amyloid A

Among the environmental factors, exposition to insecticides, mould and bacterial infections carried by *Mycobacterium tuberculosis* and *Propionibacterium acnes* (*P acnes*) seems to be the most frequent external triggers.^82^ *P acnes,* in particular, has been detected in granulomas of the epiretinal membranes in patients affected by ocular sarcoidosis^83^ (Figure 3).

The causative antigen might initiate a cross‐reacting immune response: antigen‐presenting cells (APCs) release TNF‐α, IL‐12, IL‐15, IL‐18, macrophage inflammatory protein 1 (MIP‐1), monocyte chemotactic protein 1 (MCP‐1) and granulocyte macrophage colony‐stimulating factor (GM‐CSF). Then, CD4+ T cells interact with APCs and mainly polarize in Th1 cells initiating the formation and maintenance of granulomas by secreting predominantly IL‐2 and IFN‐γ, consequently amplifying the immune response^84^ (Figure 3).

Eventually, the resulting lesions are the sarcoidal granulomas, structured masses composed of macrophages and their derivatives, epithelioid cells, giant cells and T cells which can persist, resolve or lead to fibrosis.^85^

Th1 immune polarization has been evidenced both by bronchoalveolar lavage (BAL) studies and indirectly by therapeutic evidence in which Th‐1 promoting treatments, such as IFN‐α, IFN‐γ and IL‐2, have been linked with new or relapsing sarcoidal lesions.^86^, ^87^

Among the adaptive immune effectors in granulomas formation, also Th17 cells display a key role, both in the classical variant of IL‐17‐secreting cells and through the Th17.1 phenotype which are IFN‐γ‐producing lymphocytes.^88^ Interestingly, an elevated expression of IL‐17 receptor (IL‐17R) on CD8+ T cells in peripheral blood has been found in patients with ocular sarcoidosis compared with healthy controls suggesting a role of IL‐17 signalling in sarcoidal ocular involvement^89^ (Figure 3).

Furthermore, in vitro studies have demonstrated a deficient function of Tregs in inhibiting granuloma formation despite the accumulation of CD4+CD25+FOXP3+ T cells in the periphery of granulomas^90^ (Figure 3).

Although studied to a lesser extent, the innate immune system plays its part in macrophage activation and granuloma formation: enhanced responses to TLR2 stimulation with induction of TNF‐α have indeed been detected in blood cells of patients with sarcoidosis.^91^

Progressive accumulation of serum amyloid A (SAA) within sarcoidal lesions may drive chronic disease by becoming a nidus for granuloma development and participating in feed‐forward amplification of local Th1 responses towards pathogenic tissue antigens through TLR2^92^ (Figure 3).

Clinical features of sarcoidosis depend on the location and extent of inflammation ranging from the absence of symptoms to life‐threatening consequences including respiratory insufficiency, blindness, severe neurological disease and cardiac death.^93^ Particularly, sarcoidosis is one of the leading causes of inflammatory ocular involvement.^94^

Ocular disease may present in up to 50% of patients with sarcoidosis and may progress to severe visual impairment.^95^ In fact, blindness occurs in 10% of sarcoidosis patients and is mainly attributed to cystoid macular oedema, which develops as a complication of the inflammatory alterations in the posterior segment of the eye.^94^

The most common ocular manifestations of sarcoidosis are uveitis (30%–70%) and conjunctival nodules (40%).^96^ Uveitis is a frequent and early feature of sarcoidosis and it can develop acutely, often in the context of Löfgren's syndrome (a combination of lymphadenopathies, erythema nodosum, fever and arthritis).^96^ Heerfordt syndrome is characterized by uveitis, parotitis, fever and facial nerve palsy. In chronic forms, sarcoidosis can affect any ocular tissue including the orbit and the adnexa.

Anterior uveitis is the most common ocular lesion and typical findings include mutton‐fat keratic precipitates, iris and trabecular nodules and white clumps of cells in the inferior anterior vitreous.^97^ Interestingly, anterior uveitis is more typical in black patients, whereas the more severe posterior location is most commonly observed in elderly female patients.^97^

The clinical manifestation of sarcoidal uveitis ranges from no symptoms to acute signs of inflammation: pain, photophobia, lacrimation or redness. The most frequent complications of chronic anterior uveitis are cataract, secondary glaucoma caused by extensive peripheral anterior synechia, cystoid macular oedema and band keratopathy.

When the posterior segment is affected, peri‐phlebitis and perivenous exudates may develop, traditionally named ‘candle wax drippings’.^98^ These lesions are multiple small round chorioretinal lesions typically observed in elderly white patients and are associated with frequent occurrence of cystoid macular oedema and visual loss. Frank choroidal granulomas might also be observed as white masses on the optic nerve, retina and the choroid.

Posterior segment involvement in sarcoidosis is of interest because it is frequently associated with neurological involvement (including include optic nerve disease, cranial nerve palsies, encephalopathy and disorders of the hypothalamus and pituitary gland).^95^ Retinovascular disease, instead, particularly arterial retinal macro‐aneurysms, seems to be related with severe cardiovascular disease.^95^

Intermediate uveitis with areas of snowball infiltrates may be occasionally encountered.^95^ This type of uveitis may precede more severe posterior segment changes such as retinal vasculitis and optic disc swelling.^95^ Intravitreal snowball opacities are characteristic but not specific for sarcoidosis and therefore, the differentiation from other types of intermediate uveitis might be difficult.^95^

Interestingly, an increased expression of TNF‐α, IL‐2 and IFN‐γ has been detected peripherally in supernatants of activated peripheral blood lymphocyte cultures in patients affected by sarcoidal intermediate uveitis suggesting, even in this location, a Th1 polarization.^99^

Considering the whole spectrum of sarcoidal ocular lesions and the potential severity of ocular involvement, differential diagnosis with other disorders is essential in order to establish a prompt treatment and conjunctival granulomas should always be sought for since they represent an easily accessible site for retrieving a biopsy specimen.^100^

In summary, the formation of the granuloma represents the primary hallmark lesion in sarcoid uveitis. This suggests a profound link between the innate system whose exponents are SAA‐secreting macrophages and the adaptive system, represented by Th1, Th17 and Th17.1 cells. The latter are responsible for the maintenance of the granuloma through the synthesis of IFN‐γ along with a qualitative deficiency of Tregs, incapable of curbing the uncontrolled immune reaction.

## 6 CONCLUSIONS

Inflammatory rheumatic diseases play an important role in ocular involvement. Immune‐mediated uveitides, in particular, are warning signs of underlying systemic diseases and may lead to significant visual limitation, chronic sufferance up to visual loss when the posterior segment of the eye is affected. These aspects cause a significant socio‐economic burden and deeply impact on patients’ quality of life.

The awareness of a potential severe ocular involvement might provide insights into targeted therapeutic approaches and personalized treatments for each patient.

A multidisciplinary approach, involving the ophthalmologist and the rheumatologist, is a condition easy to realize in order to improve the diagnostic rate, especially for complex and severe cases. Further clinical trials should include uveitis endpoints to offer the rheumatologic patients the optimal treatment.

## CONFLICT OF INTEREST

The authors declare no conflict of interest.

## AUTHOR CONTRIBUTIONS

EH conceived and designed the study, wrote the draft and submitted the manuscript. CAC, EG, GP, CS, EOK, VS and MC contributed to the acquisition of data and to the draft organization and approved the final manuscript.
